# Mohs micrographic surgery: a review of indications, technique, outcomes, and considerations^☆^^☆☆^

**DOI:** 10.1016/j.abd.2020.10.004

**Published:** 2021-03-24

**Authors:** Guilherme Canho Bittner, Felipe Bochnia Cerci, Elisa Mayumi Kubo, Stanislav N. Tolkachjov

**Affiliations:** a Dermatology Service, Universidade Federal de Mato Grosso do Sul, Campo Grande, MS, Brazil; b Private Practice, Campo Grande, MS, Brazil; c Dermatology Service, Hospital de Clínicas, Universidade Federal do Paraná, Curitiba, PR, Brazil; d Private Practice, Curitiba, PR, Brazil; e Post-graduate Program – Internal Medicine and Health Sciences, Universidade Federal do Paraná, Curitiba, Brazil; f Private Practice, Porto União, PR, Brazil; g Epiphany Dermatology, Dallas, TX, United States

**Keywords:** Carcinoma, basal cell, Diagnostic techniques, surgical, Minor surgical procedures, Mohs surgery, Neoplasms

## Abstract

Mohs micrographic surgery is a specialized form of skin cancer surgery that has the highest cure rates for several cutaneous malignancies. Certain skin cancers can have small extensions or “roots” that may be missed if an excised tumor is serially cross-sectioned in a “bread-loaf” fashion, commonly performed on excision specimens. The method of Mohs micrographic surgery is unique in that the dermatologist (Mohs surgeon) acts as both surgeon and pathologist, from the preoperative considerations until the reconstruction. Since Dr. Mohs’s initial work in the 1930s, the practice of Mohs micrographic surgery has become increasingly widespread among the dermatologic surgery community worldwide and is considered the treatment of choice for many common and uncommon cutaneous neoplasms. Mohs micrographic surgery spares the maximal amount of normal tissue and is a safe procedure with very few complications, most of them managed by Mohs surgeons in their offices. Mohs micrographic surgery is the standard of care for high risks basal cell carcinomas and cutaneous squamous cell carcinoma and is commonly and increasingly used for melanoma and other rare tumors with superior cure rates. This review better familiarizes the dermatologists with the technique, explains the difference between Mohs micrographic surgery and wide local excision, and discusses its main indications.

## Introduction

**Figure 1:**
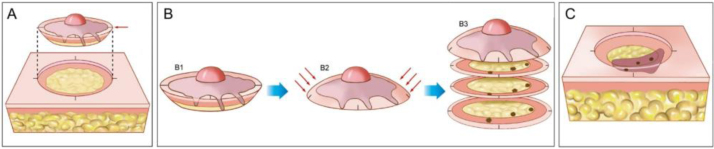
Mohs micrographic surgery. (A), Demarcation of the visible tumor and excision with a 1–2-mm margin. The incision is made with the scalpel angled at 45-degrees, which allows flattening the surgical margins on the same plane. (B), (1) Surgical specimen. The surgical margins that must be examined correspond to all lateral and deep external areas of the fragment. (2) The red arrows indicate the “flattening” of the margins to the same plane. (3) After the fragment is frozen in the cryostat, “horizontal” histological sections are performed allowing the analysis of 100% of the lateral and deep margins. The three dark spots correspond to the “roots” of the tumor seen on microscopic examination. (C), The remaining tumor “roots” are excised for further analysis under the microscope.

**Figure 2:**
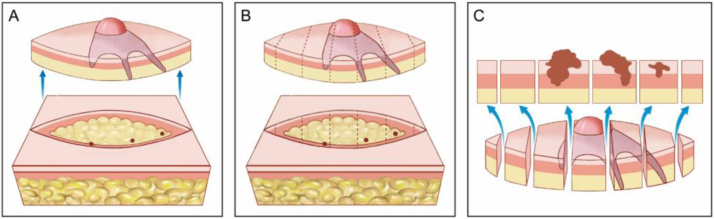
Wide local excision. (A), Elliptical excision with a wide surgical margin around the tumor. (B), Surgical specimen. On the upper drawing, the dotted lines indicate how the fragment is sectioned, similar to a loaf of bread (“bread loaf” analysis). These sections represent only about 1% of the surgical margins and may fail to see the tumor roots during microscopic examination. The three dots correspond to the tumor roots that “were left” in the patient, but were not seen in the microscopic examination because they were not included in the examined histological sections. (C), Correlation between material from surgical excision and detection of the tumor in longitudinal sections. The blue arrows indicate the histological sections that are seen in “bread loaf” analysis. Note the large amount of unexamined margins (bottom drawing) in this method.

Mohs micrographic surgery (MMS) is a specialized form of skin cancer surgery that has the highest cure rates for several cutaneous malignancies.^1^, ^2^, ^3^, ^4^, ^5^ The technique allows complete histopathological analysis of the peripheral and deep surgical margins and minimizes tissue removal, sparing normal tissue (Fig. 1). Certain skin cancers can have small extensions or “roots” that can be missed if an excised tumor is serially cross-sectioned in a “bread-loaf” fashion, commonly performed on excision specimens (Fig. 2).^6^, ^7^

Approximately 75% of all nonmelanoma skin cancer (NMSCs) are basal cell carcinoma (BCC), whereas cutaneous squamous cell carcinoma (SCC) represent approximately 20%, and the remaining are melanomas (4%) and other rare tumors.^8^ MMS is the standard of care for selected BCCs and SCCs and is commonly and increasingly used for melanomas and other tumors.^5^

MMS is unique in that the dermatologist (Mohs surgeon) acts as both surgeon and pathologist allowing precise correlation of the surgical wound with the Mohs map.^6^ Once clear margins are obtained; the Mohs surgeon may perform same-day reconstruction unless multi-specialty referrals are needed.

## History

Dr. Frederic E. Tromovitch (1932-1990), started his work in what he called “chemosurgery” in the 1930s, but his findings were not published until 1941.^9^ In 1936, he started using this technique in patients deemed incurable. He would apply a 20% zinc chloride paste on the skin cancer in vivo and leave it overnight to fixate the skin. While painful for the patient, this was quite effective in preserving the microscopic anatomy of the skin.^2^, ^6^

In the 1960s, Dr. Theodore Mohs (1910-2002), first published an article using the fresh tissue technique on tumors located on other parts of the body (Dr. Mohs first did it in 1953 but it was not published).^10^ The modifications by Dr. Tromovitch of Dr. Mohs’s technique enabled the procedure to be done in a single day.^6^ Mohs presented his data, a cohort of 70 basal cell carcinoma (BCCs) on the eyelid removed with the fresh-frozen technique, in 1969. The following year, Dr. Theodore Tromovitch published a series of an additional 75 cases successfully treated with the fresh-frozen technique. These findings further validated the efficacy of this method.^2^ The fresh-frozen tissue technique quickly became the standard of care, and in 1987, the American College of Chemosurgery was renamed as the American College of Mohs Surgery and Cutaneous Oncology to reflect this practice.^2^

Since Dr. Mohs’s initial work in the 1930s, the practice of MMS has become increasingly widespread among the dermatologic surgery community worldwide and is now considered the treatment of choice for many common and uncommon cutaneous neoplasms.^11^, ^12^, ^13^, ^14^, ^15^

## MMS training

In the US and Brazil, only dermatology-trained physicians are allowed to apply for a fellowship in MMS, and a minimum of 12 months of supervised training under the program director is required. The fellow must review all pathology from the surgical cases done in the training program. Some practical components of the training program are different between the two countries, especially regarding the number of cases (much higher in the US).

The main objective of the training is to become able to evaluate the histopathology of the section, especially tumor-free margins (normal skin). Mohs surgeons must also have expertise in skin reconstruction, repairing most of the cases, to optimize functional and aesthetic outcomes.

In Brazil, the Brazilian Society of Dermatology has a certification in micrographic surgery for the dermatologic surgeons that fulfill the training program.^16^ Most programs are affiliated to a residency program. In the US, MMS training programs are accredited by the Accreditation Council for Graduate Medical Education (ACGME) and the American College of Mohs Surgery (ACMS). While some fellowship programs, private and academic practices, were originally accredited by the ACMS, all US-based fellowships now have to be ACGME approved, while a few international fellowships still carry an ACMS approval but not ACGME. Most of these fellowship directors were trained in the US and started fellowships in other countries. Other societies like the American Society for Dermatologic Surgery (ASDS) and the American Society for Mohs Surgery (ASMS) have dermatologic surgical programs but are not formal Mohs fellowships. The ASDS also has fellowships in cosmetic surgery that may share the same fellowship directors as the ACMS Mohs surgery programs.

In October of 2018, the American Board of Medical Specialties (ABMS) approved the American Board of Dermatology (ABD) for a new board certification in Micrographic Dermatologic Surgery (MDS).^17^ According to ABD, candidates for the MDS subspecialty certification must:

1 Possess a current, valid, full, and unrestricted license to practice medicine or osteopathy in at least one state or province within the United States or Canada;
2 Hold primary certification in general dermatology from ABD;
3 Be up to date in Maintenance of Certification (MOC) if certification by ABD is time-limited;
4 Demonstrate experience in the subspecialty by successfully completing the ACGME-accredited Micrographic Surgery and Dermatologic Oncology (MSDO) fellowship OR during an initial 5-year practice pathway eligibility period only, attesting to practicing micrographic surgery; and
5 Pass the MDS certification examination.^17^

While it is not fully known if the ACMS will accept those who have passed the MDS examination and met the aforementioned requirements or if other societies will continue to keep a separate certification and meetings, after the 5-year period, all candidates to become Mohs surgeons must train in an ACGME-accredited Mohs surgery fellowship and meet the MDS requirements. In addition, private-practice fellowships will likely be decreasing in numbers and limited in the upcoming years, as a sponsoring institution with a dermatology residency is now required for a fellowship program.

## Preoperative considerations

### Prophylactic antibiotics

Most patients do not require prophylactic antibiotics. However, in some cases it is indicated for one of the following reasons: reduce the likelihood of surgical site infection, risk of endocarditis, and risk of prosthetic infection. To reduce surgical site infection, the main indications consist of surgeries on the lower extremities (mainly legs) and groins, wedge excision of ear or lip, flaps on the nose, and grafts.^18^

Antibiotic choices include oral cephalexin 2 g or, for penicillin-allergic patients, clindamycin 600 mg. If the preoperative antibiotic dose was missed inadvertently, the medication may be given up to 2 -hs after the procedure.^18^ In addition, there are publications showing success with clindamycin mixed with lidocaine for additional intraincisional local antibiotic coverage.^19^, ^20^, ^21^

### Anticoagulants

Prior to cutaneous surgery, a discussion of whether to continue anticoagulation medications is necessary. It is important to weigh the risks and benefits of stopping medications vs continuing.^22^ In general, if the patient has a history of thrombotic events (secondary prevention), medications should be continued. If they are used for primary prevention and the risk of bleeding may significantly impair the surgical outcome, discussion with the primary care provider whether to stop or not is an option. Otherwise, the recommendation is to continue.^23^

In 2011, a study demonstrated that clopidogrel was 28 times more likely than no anticoagulation and 6 times more likely than aspirin to result in severe complications after MMS (including bleeding, hematoma, and infection). However, complications were not life-threatening.^24^ In another study with 2,790 patients, 68 underwent surgery while taking warfarin (2.4%). Intraoperative bleeding was easily controlled, and postoperative bleeding was not recorded in any of the patients.^25^ Dabigatran and Rivaroxaban did not demonstrate severe hemorrhagic bleeding.^23^, ^26^

It is also important to note that serious adverse vascular events have been reported after discontinuation of anticoagulants. In some cases, these thrombotic events have resulted in serious impairment or death.^27^ Based on the literature, medically necessary anticoagulant and antiplatelet medication should be continued during MMS. In general, bleeding complications are uncommon and easy to manage. Special attention must be paid to achieving intraoperative hemostasis. The use of pressure bandages should be considered in patients taking anticoagulants, in particular, clopidogrel and warfarin.^22^

### Pacemakers and implantable cardiac defibrillators

The use of electrocautery to achieve hemostasis is of particular concern in patients with a pacemaker, Implantable Cardioverter Defibrillator (ICD), or other implantable medical devices.^2^ A retrospective chart review of patients with pacemakers (n = 173) or ICDs (n = 13) submitted to MMS or WLE had no documented complications from electrosurgery.^28^

An in vitro study concluded that hyfrecators are safe to use in patients with defibrillators. The authors investigated safety margins and reported that for pacemakers, atrial inhibition was observed at 3 cm on maximum hyfrecator settings and 1 cm at normal use settings.^29^ A recent in vivo retrospective study did not report adverse perioperative effects in patients with implantable cardiac devices.^30^

The lack of complications associated with pacemakers and ICDs during electrosurgery is reassuring. The authors support published recommendations about techniques and precautions to optimize safety during electrosurgery in patients with cardiac devices. The care of patients with ICDs, in particular, requires special consideration.^28^ The discussion with the assistant cardiologist is important since some cardiac devices can be temporarily turned off with a magnet.^31^ Another possibility in some cases is the use of disposable electrocautery.

### Classical surgical technique

The first stage of MMS is marking the clinically evident tumor margins to be excised (usually from 1 to 2 mm). This may be done with the help of surgical magnifying glasses or a dermatoscope. After the marking, the area is cleansed with antiseptic and local anesthesia is done, typically with buffered lidocaine with epinephrine. Then, the clinically evident tumor is removed (debulking) with either a blade (sharp debulking) or a curette, being careful not to erase surgical markings. Next, first stage margins are removed with the blade oriented at a 45-degree angle to the skin.^5^ Beveling of the scalpel blade allows for proper alignment of the peripheral edges of the tissue specimen during the preparation of the histopathological specimen. During first stage excision, markings are done at the 12, 3, and 6 o’clock positions for further orientation allowing location of residual tumor (in case the residual tumor is detected during microscopic examination).

Once the specimen is entirely removed, hemostasis is obtained. Some surgeons inject an additional long lasting anesthetic (bupivacaine 0.5%) to prevent local pain while the patient awaits the laboratory processing of the specimen and the reading of the histologic slides.^32^ While tissue is processed (20-min to 1-h), the patient awaits, with a bandage on the wound, in a waiting room or in the surgical room.

**Figure 3:**
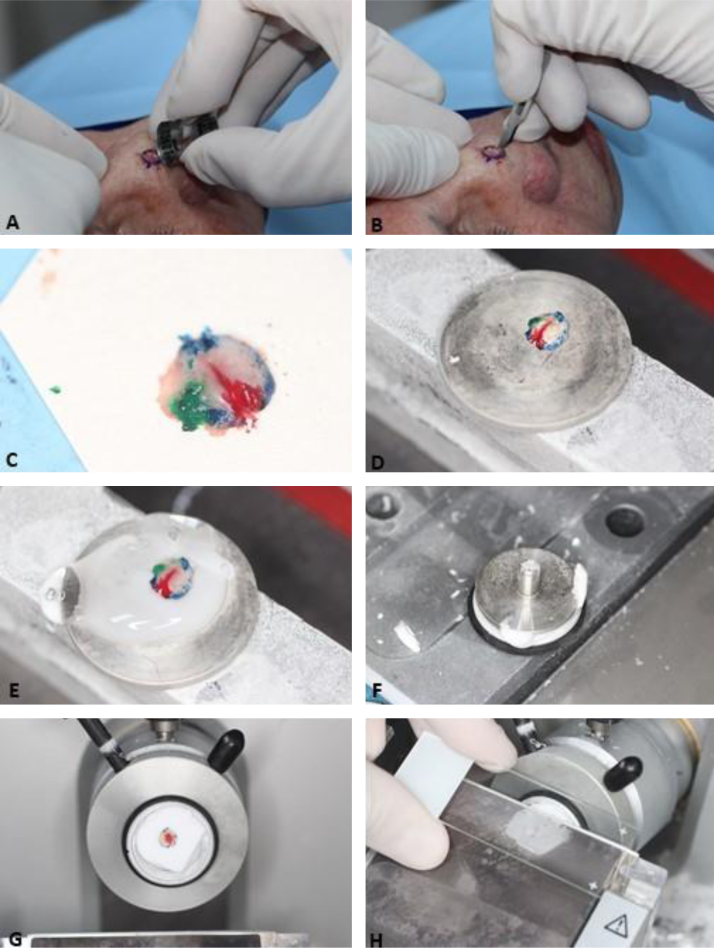
Surgical Technique. (A), Clinically evident tumor is removed (debulking) by saucerization. (B), First stage margins are removed at a 45-degree angle. (C), Surgical specimen flattened (lateral margins are on the same plane as the deep margin) and colored with different dyes for orientation. (D), Surgical specimen frozen on a metal plate. (E–F) A specific gel called Optimum Cutting Temperature (OCT) is added to the metal plate to create the tissue block. (G), Tissue block ready for sectionning. (H), The tissue block is sectioned on a microtome and several sections are placed on the histologic slides. Slides are then stained and coverslipped (not shown in the figure) before microscopic examination.

The first step of tissue processing is to flatten the surgical specimen, ensuring that the beveled peripheral margins are on the same plane as the deep margin. Tissue may be additionally flattened with relaxing incisions. Next, markings are inked with different colors (chromacoding). For correlation with the surgical defect, a two-dimensional map of the lesion and corresponding area is drawn using the same colors to identify each marking. Another option is to create a digital Mohs map by photographing the wound area and coloring the markings on the tablet/computer.^33^, ^34^ The choice of mapping is influenced by surgeon preference or training and added to the clinic protocol. The specimen is then frozen surrounded by a specific gel called Optimum Cutting Temperature (OCT) and fixed to a chuck. Finally, the tissue block is sectioned on a microtome and several sections are placed on the histologic slides (Fig. 3). These are then stained with hematoxylin and eosin (although other stains like toluidine blue are sometimes used) and coverslipped. Histologic slides are then examined under the microscope by the Mohs surgeon. Technique artifacts can happen if the steps are not adequately followed, including poor coloration, curled sections, slides without epidermis (not flattened enough) or subcutaneous tissue (insufficient freezing time), freezing artifacts, or tumor floaters (which are fragments of tumor tissue which have been displaced into tumor-free areas during flattening of the specimen).^35^ In addition, errors can occur because of tissue that “drops out” during processing, improper mapping, and correlation, when surgeons do not appropriately excise sufficient potentially-normal tissue to ensure clearance of all tumor tissue and non-contiguous tumors.

If residual tumor is noted at the microscope (positive margins), the process is repeated. A new 1–2 mm margin is again taken only around the area compromised and mapped positively during microscopic evaluation. If only the deep margin is involved, the second stage can be taken in that area without enlarging the diameter of the defect. Careful handling is important to maintain orientation. In addition, it’s important to take tissue fully around the mapped tumor positive area to limit the chance of sampling error of the second and subsequent stages.^36^, ^37^ Once margins are tumor-free, the Mohs surgeon or multi-specialty colleagues can safely reconstruct the wound.

### Variations on the surgical technique

1 – Incision degree angle: Some Mohs surgeons incise the skin with the blade angled at 90-degrees instead of the traditional 45-degree beveled angle. Although the 90-degree angle variation requires more laboratory work to flatten or “relax” the surgical specimen, it is an alternative as long as the entire surgical margin can be flattened to the same section plane.^38^ A potential pitfall of this is “pushing through” tumor tissue or creating tissue gaps when making relaxing cuts. However, once the surgery is done, there is no need to de-bevel prior to reconstruction.

2 – Methods for flattening, freezing, and embedding the specimen: Options include in vivo intraoperative relaxing incisions, heat extractor flattening at the cryostat, freezing on a glass slide, the Miami Special technique, cryomold, the Cryo-Embedder*^TM^*, and the Rio de Janeiro technique.^39^, ^40^, ^41^, ^42^, ^43^ Regardless of the chosen method, the main objective is to prepare high-quality slides.

3 – Staining variations: Instead of hematoxylin and eosin, some Mohs surgeons use toluidine blue.^44^ When using toluidine blue, the typical finding is metachromasia defined as a magenta hue, which typically surrounds BCC nests.^45^ In SCC, this finding tends to be less intense.^46^

## Wide local excision compared to MMS

Wide Local Excisional (WLE) specimens sent to the pathology laboratory for evaluation of margins are processed in a cross-sectional or “bread-loaf” manner. Representative tissue is sliced vertically at 2- to 4-mm intervals to check for tumor presence at the surgical margin.^6^ The amount of tissue analysed depends on the number of sections obtained. Typically, less than 1% to 2% of the specimen margin is evaluated. Sampling error will occur if the intervals of the sections miss extensions of the tumor, which may penetrate between the sampled sections.^5^, ^7^ Figure 1, Figure 2 compare the histologic evaluation of WLE versus MMS.

It is extremely important to mention that WLE with frozen section analysis is not the same procedure as MMS. In WLE with frozen section analysis, the evaluation of margins follows the same process as the traditional WLE (bread-loaf technique) so tumor compromised margins may be missed.^6^

The Tubingen Torte Technique, the Munich Method, and excision with *en face* margin analysis also differ from MMS. Tubingen Torte Technique is performed on paraffin-embedded sections and separates the lateral margins from the deep margins for histopathological analysis.^47^ During histologic sectioning, the external lateral margins are sectioned on the same plane and the deep margins from the bottom up.^48^ In the Munich method, there is no debulking; the lateral and deep margins are not flattened into one plane before sectioning and the excision margins are perpendicular to the skin surface.^49^ Horizontal sections are obtained in the cryostat (every 150 microns) from the bottom up to the epidermis surface.^50^ Excision with *en face* margin analysis is similar to the Munich method, but the tissue is sectioned from the epidermis to the bottom and the deep margin may not be fully evaluated.^51^, ^52^ These methods aim to achieve a complete circumferential peripheral and deep margin assessment but are outside the scope of this article. Detailed descriptions of each method have been published.^47^, ^48^, ^49^, ^50^, ^51^, ^52^ As with any procedure, clearance and recurrence rates of these techniques may be dependent on the training and experience of the provider and proper tumor selection for the appropriate treatment technique. However, the ability of 100% deep and peripheral tumor margin assessment by the same doctor acting as surgeon and pathologist at the same time allows for the highest published cure rates and accurate clinicopathologic correlation in real-time.

### Indications of MMS

The indications of MMS follow the Appropriate Use Criteria (AUC) guidelines created by the American Academy of Dermatology, American College of Mohs Surgery, American Society for Dermatologic Surgery Association, and American Society for Mohs Surgery to help clinicians select the most appropriate neoplasms for treatment with MMS.^2^, ^53^, ^54^ In 2016, a Brazilian guideline was written with similar recommendations.^15^

**Figure 4:**
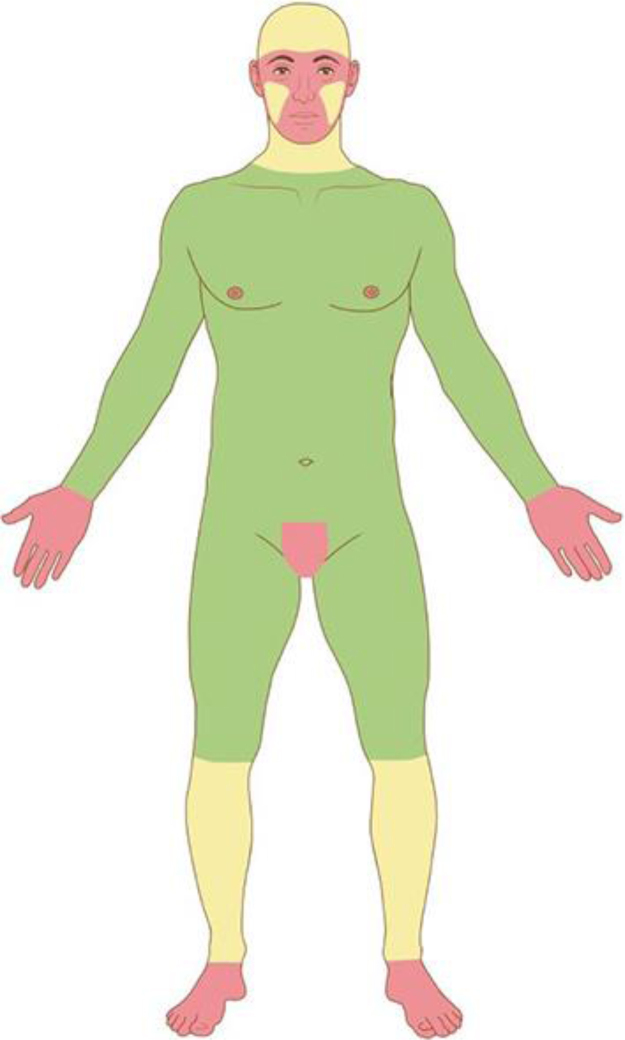
Anatomic risk areas. Red: High-risk areas. central face, eyelids, eyebrows, periorbital skin, nose, lips, chin, mandible, preauricular and postauricular skin, temple area, ear, genitalia, hands, and feet. Yellow: Medium-risk areas. cheeks, forehead, scalp, neck, and pretibial. Green: Low-risk areas. Trunk and extremities (excluding hands, feet, nail units, pretibial area, and ankles).

**Figure 5:**
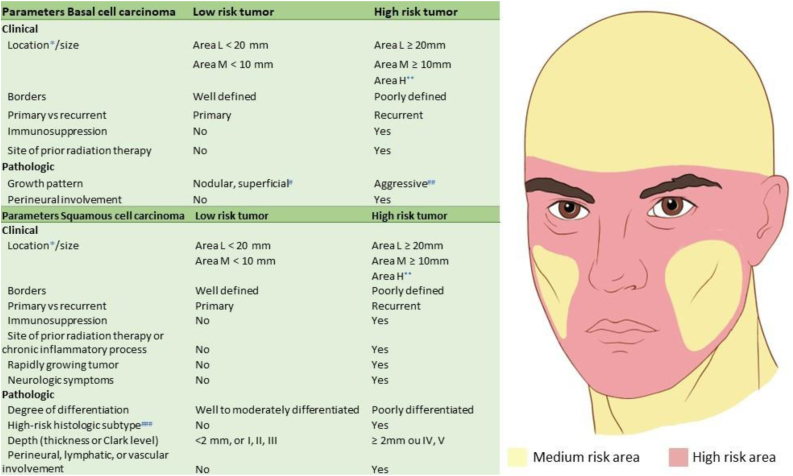
Mohs micrographic indications for BCC and SCC. (*) For further details of risk areas, check Fig. 4. (**) Area H constitutes high-risk based on location, independent of size. (#) Other low-risk growth patterns include keratotic, infundibulocystic, and fibroepithelioma of Pinkus. (##) Having morpheaform, basosquamous (metatypical), sclerosing, mixed infiltrative, or micronodular features in any portion of the tumor. (###) Acantholytic and desmoplastic subtypes.

Based on the characteristics of an individual tumor, anatomic location (Fig. 4), and unique patient characteristics, the tumor is considered a low or high-risk tumor. Basically, the high-risk BCC, high-risk SCC, and the rare NMSC (DFSP, mucinous carcinoma, etc.) should be referred for MMS (Fig. 5); as well as Melanoma In Situ (MIS) in high-risk areas.^2^, ^5^, ^15^, ^53^^,^^54^ Invasive melanomas are increasingly being treated with MMS demonstrating cure rates comparable to and often better than WLE.^55^, ^56^

### MMS safety

MMS is traditionally performed under local anesthesia. In the United States, where the technique is performed frequently, only on rare occasions MMS is performed under conscious sedation or general anesthesia.^57^, ^58^, ^59^ Local anesthesia is the safest method for the patient because MMS can take several hours.^5^, ^57^, ^60^, ^61^, ^62^ Measures to minimize discomfort of local anesthesia should be taken. Overall, MMS is considered a safe procedure due to the local anesthesia and avoidance of the risks of general anesthesia. Patients that may be considered “poor surgical candidates” for the operating room with general anesthesia are typically able to undergo MMS. A large 23-center prospective study evaluated Adverse Events (AE) associated with MMS surgery. Minor postoperative complications and serious AE were reported in 0.72% and 0.02% of cases, respectively. The most common complications included infections, bleeding, or impaired wound healing. Only 4 of 20,821 cases studied required admission to the hospital, constituting 2.7% of all adverse events and 0.02% of all procedures, and there were no cases of permanent disability or death. Among patients that were hospitalized, no cases happened during surgery, all were in the postoperative period due to local infection, and all were treated with intravenous antibiotics. None of the hospitalized patients had life-threatening events or death.^57^ Other similar studies disclosed no serious complications after cutaneous surgery that required hospitalization.^60^

In a study with 3,937 patients comparing major complications from MMS performed in office-based versus hospital-based settings the results showed that procedures were equally safe in both settings. The authors emphasized that dermatologic surgeons may continue to routinely perform these procedures in an outpatient office-based setting.^63^

Most complications following MMS surgery are managed by Mohs surgeons in their offices, minimizing the need for utilization of additional health care resources.^22^, ^57^, ^60^, ^64^ This is important in times when personal protective equipment and hospital beds are scarce and rationed, such as during international pandemics like COVID-19.

The majority of tumors treated with MMS are removed in one or two stages and are repaired with primary closure, which is associated with fewer complications and less postoperative pain compared with other repairs.^60^ Patients, in general, have low peak pain levels following MMS and reconstruction and are highly satisfied with their pain control.^22^, ^60^, ^64^

### MMS efficacy

Table 1 demonstrates MMS efficacy for different cutaneous malignancies.^1^, ^3^, ^65^, ^66^, ^67^, ^68^, ^69^, ^70^, ^71^, ^72^, ^73^, ^74^, ^75^, ^76^, ^77^, ^78^, ^79^, ^80^, ^81^, ^82^, ^83^, ^84^, ^85^

**Table 1:** Comparison of cure rates after 5-years for different cutaneous malignancies treated with Mohs surgery or wide local excision.

Tumor	Cure rates (%)	
	Mohs micrographic surgery	Wide local excision
Basal cell carcinoma^1^, ^3^, ^65^	99 (primary)	87–96 (primary)
	90–93 (recurrent)	83 (recurrent)
Squamous carcinoma^65^, ^66^, ^67^	92–99 (primary)	92–95 (primary)
	90 (recurrent)	76 (recurrent)
Melanoma in situ^68^, ^69^	98	83–85
*Dermatofibrosarcoma protuberans*^70^, ^71^	98–100	80–88
Atypical fibroxanthoma^76^	98	91
Microcystic adnexal carcinoma^72^	94–96	40–83
Sebaceous carcinoma^72^, ^73^, ^74^, ^75^	94	64–89
Extramammary Paget desease^77^	92	78

Adapted from: Tolkachjov SN et al., 2017.^5^

Other tumors such as leiomyosarcoma, melanoma, hidradenocarcinoma, Merkel cell carcinoma, mucinous carcinoma, and porocarcinoma have shown higher cure rates with the use of MMS.^78^, ^79^, ^80^, ^81^, ^82^, ^83^, ^84^, ^85^

### Tissue sparing with MMS

Conservation of healthy tissue has always been a matter of interest in skin cancer surgery. MMS has been shown in multiple studies to be tissue-sparing when compared to standard excision.^86^ A recent study with infiltrative BCCs demonstrated that MMS saved 46% of healthy tissue when compared to standard surgery.^87^ Another study demonstrated significant preservation of healthy tissue (56%–86%) for facial BCCs near free margins when compared to the recommended standard excisional margins.^88^ Similarly, Gniadecki et al., showed that MMS leads to respectively 43% and 45% smaller defects compared to standard excisions of primary BCC (4-mm margins) and high-risk, recurrent BCC (6-mm margins).^89^ A small randomized trial for nodular BCCs <1 cm reported that defect size after MMS was significantly smaller (p < 0.001) than after standard excision (116.6 vs. 187.7 mm^2^).^86^, ^88^

## Basal cell carcinoma

BCC is the most common human malignancy.^2^ Although it rarely causes major complications, such as metastasis or death, BCC can be locally destructive invading deeper tissues such as muscle, cartilage, and bone.^90^ The most common features associated with significant subclinical extensions are high-risk location, recurrent tumor, aggressive subtype, and tumor size >10 mm.^91^, ^92^ A recent study that evaluated margins (in mm) required for primary BCC clearance showed that the most significant factors related to larger margins were superficial, micronodular, infiltrative, and morpheaform subtypes; whereas clinically well-defined tumors were associated with smaller margins.^93^

The most clinically relevant stratification to guide the management of patients with BCC is the differentiation between low-risk versus high-risk tumors for recurrence (Fig. 5).^53^ MMS has been shown to have superior cure rates in primary and recurrent BCCs.^1^, ^3^ The recurrence rates are outlined in Table 1. It is also an efficient and cost-effective procedure as the treatment of choice for high-risk BCCs and those in cosmetically sensitive locations.^5^, ^15^, ^94^

## Squamous cell carcinoma

SCC is the second most common form of skin cancer. Similarly to BCC, SCC is increasing in incidence throughout the world.^54^ They are typically graded as well-, moderately-, or poorly-differentiated neoplasms. Unlike BCCs, SCCs have significant metastatic potential.^2^, ^54^, ^95^ A 10-year retrospective cohort study showed that patients with SCC had a 3.7% incidence of metastasis and 2.1% risk of disease-specific death.^96^ In the US, aggressive or high-risk SCCs are estimated to result in approximately 8,000 cases of lymph node (LN) metastases and 3,000 deaths each year.^95^ As with BCC, published risk factors like tumor location and patient immunosuppression due to hematologic malignancies, immunosuppressive medications, organ transplantation, or genetic or acquired immunosuppressive diseases like Human Immunodeficiency Virus (HIV) are also associated with higher recurrence and metastatic rates, and these patients are most likely to benefit from MMS for SCC.^97^ Multivariate analysis of tumor characteristics found that 5-high-risk factors were statistically independent prognostic factors: poor differentiation, perineural invasion, tumor diameter >2 cm, invasion of subcutaneous fat and ear, temple, or genital location.^96^ Brigham and Women’s Hospital (BWH) uses 4 of these high-risk factors (poor differentiation, perineural invasion, tumor diameter >2 cm, and subcutaneous fat invasion) to create a staging system, where: T0 is in situ SCC; T1 – zero risk factors; T2a – 1 risk factor; T2b – 2–3 risk factors; T3 – 4 risk factors or bone invasion.^96^

It cannot be overemphasized that the best chance for SCC cure is in the complete surgical removal of the initial lesion, as all tumor treatment options are less successful for persistent or recurrent tumors than for primary tumors. Once an SCC has recurred, the risk of spread to regional LN and distant metastases increases significantly. High-risk tumors (Fig. 5) are best treated with MMS.

In a cohort study, 647 patients treated with MMS alone for high-risk SCC, there were 19 local recurrences (2.9%), 31 nodal metastases (4.8%), 7 distant metastases (1.1%), and 7 disease-specific deaths (1.1%). They experienced the lowest rates of local recurrence, nodal metastasis, and disease-specific death published thus far for high-risk SCC.^98^

SCCs with a single high-risk feature have a low risk for metastasis and death but an increased risk of local recurrence. A recent study comparing outcomes for these intermediate-risk tumors, BWH T2a, found that MMS provided improved outcomes when compared to WLE with permanent sections.^99^

Treatment of SCC with MMS has shown superior cure rates than WLE, and local recurrences occur less frequently when SCC is treated by MMS, demonstrating to be a highly effective modality in the treatment of these tumors.^5^, ^98^

## Melanoma in situ

Although MMS has been increasingly used for invasive melanomas in the US, we will limit our discussion to MIS. The initial studies of the successful use of MMS for primary cutaneous melanoma date from the late 90s. At the time, surgical specimens were performed with H&E stain.^68^ Immunohistochemistry (IHC) using the melanoma antigen recognized by T cells-1 (MART-1) has been used now for many years and has increased the accuracy of margin examination with the frozen section processing of MMS and is the recommended method for those who treat melanomas with MMS.^100^ Other stains like MITF and Sox10 have also been used, and others are being studied.

In a prospectively study of 1,120 MIS (1.072 patients) treated with MMS 3 patients (0.3%) had local recurrence and 3 had distant metastases. In all, 86% of MIS were successfully excised with a 6-mm margin; and 98.9% with 9-mm.^101^ In Brazil, the cost of MART-1 limits the use of MMS to treat MIS.

## Dermatofibrosarcoma protuberans

DFSP is a rare fibroblastic tumor with a slow infiltrative growth that rarely metastasizes.^102^ DFSP is known for its extensive subclinical spread, therefore meticulous margin control is recommended.^102^ For WLE, recommended margins are 3-cm whereas MMS initial margins are at least 1-cm.^103^ One of the first studies using MMS for DFSP reported no recurrence after an average follow-up of 3.4 years in a series of 20 cases.^104^

A more recent study with long-term follow-up comparing WLE versus MMS demonstrated a recurrence rate of 30.8% and 3%, respectively. Furthermore, although preoperative tumor sizes were similar, MMS resulted in smaller defects allowing more primary closure (73%), whereas tumors treated with WLE required flaps or grafts in52% of cases.^105^

Ideally, MMS for DFSP should be performed with the IHC marker CD34 to better detect tumor cells, especially in recurrent cases where it may be difficult to distinguish scar from tumoral tissue.^102,103^ When available, MMS (or another method of complete histologic margin control) is the recommended treatment for DFSP due to its higher cure rates and reduced morbidity.^105^

## Microcystic adnexal carcinoma

Microcystic Adnexal Carcinoma (MAC) is a sclerotic sweat gland carcinoma that presents as a slow-growing indurated and often hypopigmented plaque on the head and neck. It affects sun-exposed areas of older adults, usually Caucasians.^72^, ^106^ MMS has become an important indication for MAC because of its likelihood of subclinical extension.^107^ The local recurrence rate is between 17% and 60% with WLE and 4.7% to 5.7% with MMS.^72^

## Sebaceous carcinoma

Sebaceous carcinoma is derived from the adnexal epithelium of the sebaceous glands usually occurring in adults older than 60-years, on the eyelid, head and neck, and trunk. It is often seen as a pink to red-yellow papule or nodule that may bleed in a third of the cases.^51^, ^72^

A consensus of a panel of experts believes that margin control with MMS or other methods of complete circumferential peripheral and deep margin assessment are most likely to achieve tumor clearance and reduce the risk of recurrence.^51^ WLE recurrence rate is 11% to 36% after 5-years and an 18% to 30% 5-year mortality has been reported with WLE of 5- to 6-mm margins on periocular location.^73^, ^75^ Although reporting bias may distort the data, the overall recurrence rate of sebaceous carcinoma treated with MMS is 6.4% (7/109) with a metastatic rate of 3.7% (4/109).^72^

## Atypical fibroxanthoma

Atypical Fibroxanthoma (AFX) is a fibrohistiocytic tumor with a relatively high local recurrence rate but low metastatic potential. Clinically, AFX typically presents as a pink nodule on the head and neck in elderly white men, growing over several months.^76^

A systematic review of a pooled group of 907 patients with 914 tumors, demonstrated a lower recurrence rate with MMS than WLE (2.0% vs. 8.7%). Although the gap of the recurrence rate between MMS and WLE is narrow, we have to consider that 80% of AFX starts on the head and neck, hence, MMS emerges as the most suitable technique to ensure microscopically controlled excision while optimizing tissue sparing on anatomically sensitive areas.^76^

## Other cutaneous tumors

Almost all types of cutaneous malignancies have been treated through MMS over the decades, even the rare non-melanoma skin cancers, with superior results to WLE (Table 1).^82^, ^85^ Although these tumors vary in the anatomical structure of origin, they all share one crucial aspect: they are contiguous tumors, often with subclinical extension underneath the skin surface, rendering a non-MMS surgeon’s subjective measurement of the tumor margins less accurate than an MMS surgeon’s ability to assess the complete peripheral and deep margins microscopically.^5^

## Reconstruction after MMS

Reconstruction is only performed after margins are confirmed to be histologically tumor-free, ideally when the wound is freshly created rather than open or bandaged for an extended period of time prior to reconstruction.^5^ On the other hand, on excisional surgery, reconstructions are performed without knowing if the tumor was completely removed. This is the reason why NCCN does not recommend any kind of tissue rearrangement (flaps) after WLE; the flap may “hide” cancerous cells making recurrences catastrophic.^108^ In the authors’ experiences, same-day reconstruction with clear margins is preferred by patients undergoing cutaneous surgery.^109^, ^110^

**Figure 6:**
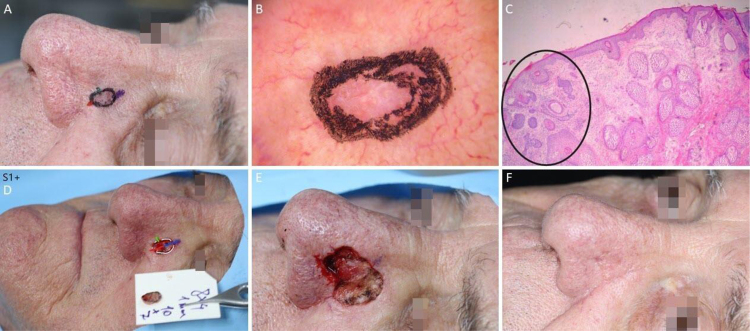
BCC with extensive subclinical extension. (A), Surgical margins of the first stage. (B), First stage margins based on dermoscopy, with fine telangiectasias and white red structureless areas. Although the figure shows many telangiectasias surrounding the first stage margin, these telangiectasias were interpreted as resulting from photodamage since they were widely distributed on his central face. After margins were clear, similar telangiectasias were still present surrounding the defect. (C), Histological section of the specimen obtained with Mohs surgery showing nests of basaloid cells adjacent to or conected to the epidermis, consistent with mixed basal cell carcinoma (Hematoxylin & eosin, ×40). (D), Digital map of the first stage. White lines indicate the presence of residual tumor. (E), Surgical wound after 6 stages, with the involvement of the left nasal wall and medial malar region. (F), 2-months postoperatively. Cheek restored with primary closure and nasal sidewall with Burow full-thickness skin graft and second intention healing.

**Figure 7:**
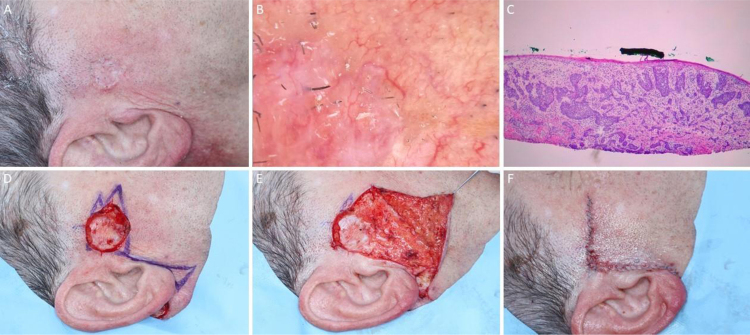
Ill-defined basal cell carcinoma on the right pre-auricular area. (A), Preoperative. (B), Dermoscopy showing arborizing vessels and shiny white, red structureless areas. (C), Histological section of Mohs surgery specimen demonstrated irregular nests of basaloid cells, consistent with infiltrative basal cell carcinoma (Hematoxylin & eosin ×25). (D) Surgical wound after 2 stages. Advancement flap design. (E), Flap undermined. Note that M-plasty was not performed. (F), Immediate postoperative.

**Figure 8:**
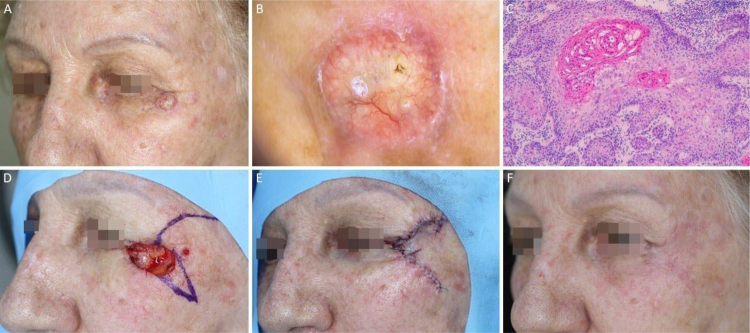
Squamous cell carcinoma on the left periorbital region. (A), Preoperative oblique view. (B), Bright whitish-yellow areas and arboriform telangiectasias. (C), Histological section of Mohs surgery specimen showing atypical keratinocytes and horn pearls, consistent with well-differentiated squamous cell carcinoma (Hematoxylin & eosin ×100). (D), Rotation flap design. (E), Immediate postoperative. (F), 2-month postoperatively.

**Figure 9:**
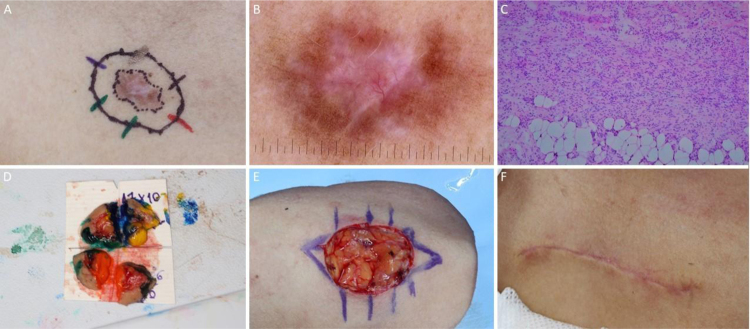
Dermatofibrosarcoma protuberans. (A), Tumor on the right infraclavicular region and first stage margins. (B), Dermoscopy showing thin and linear arboriform telangiectasias, shiny white streaks, pink amorphous area, surrounded by a pigmented network. (C) Histological section of Mohs surgery specimen showing a fusocellular proliferation in the dermis and subcutaneous tissue, consistent with dermatofibrosarcoma protuberans (Hematoxylin & eosin ×100). Preoperative immunohistochemistry was positive for CD34. (D), Surgical margins mapped with different colors. (E), Surgical wound and primary closure design. (F), 1-month postoperatively. Erythema resolved over time.

Mohs surgeons must have the ability to perform a variety of techniques when closing both simple and more complex skin defects, including complex linear closures, skin flaps, and grafts. Even large flaps, interpolation flaps, and large grafts are safely performed in the office setting under local anesthesia.^111^, ^112^, ^113^ In the US, dermatologists represent the largest specialty group performing reconstructive surgeries, performing more than 4-times the number of local flaps and more than 6 times the number of grafts as plastic surgeons, meanwhile averaging a significantly larger number of average per-physician yearly cases than any other specialist group.^114^, ^115^ In a minority and specific cases, a multidisciplinary approach with oculoplastics, plastic surgery, urology, and otolaryngology (ENT Surgeons) or head & neck oncology may be the best option for complicated cases.^116^, ^117^ Figure 6, Figure 7, Figure 8, Figure 9 illustrate MMS cases performed by the authors.

## Final considerations

MMS is an effective treatment modality for numerous cutaneous malignancies. It has achieved statistically significant superiority to WLE for the treatment of BCC, SCC, and other cutaneous tumors. Dermatologists and non-dermatologists managing cutaneous malignancies must understand MMS indications so patients can have a higher cure rate avoiding future recurrences and complications associated with their treatment.

The future of MMS is likely to see an increased application for a variety of cutaneous malignancies and increased use of noninvasive imaging like Multispectral Optoacoustic Tomography, Optical Coherence Tomography to better understand in vivo tumor characteristics.^118^, ^119^ Ex vivo fluorescence confocal microscopy is a new imaging tool that offers an attractive alternative to conventional frozen histology for the evaluation of margins in freshly excised tissue although technical issues still prevent the wide use of this technique.^120^ However, until concrete studies with long-term follow-up are performed, MMS with hematoxylin e eosin histologic evaluation will continue to be the mainstay treatment.

## Financial support

None declared.

## Authors’ contributions

Guilherme Canho Bittner: Participation in the conception and planning of the study; obtaining, analyzing, and interpreting the data; writing; approval of its final version.

Felipe Bochnia Cerci: Participation in the conception and planning of the study; analyzing, and interpreting the data; writing; critical review of the manuscript; approval of its final version.

Elisa Mayumi Kubo: Writing; approval of its final version.

Stanislav N. Tolkachjov: Obtaining analyzing and interpreting the data; critical review of the manuscript; approval of its final version.

## Conflicts of interest

None declared.
